# YOLO-GRBI: An Enhanced Lightweight Detector for Non-Cooperative Spatial Target in Complex Orbital Environments

**DOI:** 10.3390/e27090902

**Published:** 2025-08-25

**Authors:** Zimo Zhou, Shuaiqun Wang, Xinyao Wang, Wen Zheng, Yanli Xu

**Affiliations:** 1 School of Information Engineering, Shanghai Maritime University, 1550 Haigang Avenue, Pudong New Area, Shanghai 201306, China; 15155750620@163.com (Z.Z.); ylxu@shmtu.edu.cn (Y.X.); 2 Key Laboratory for Satellite Digitalization Technology, Chinese Academy of Sciences, 99 Haike Road, Pudong New Area, Shanghai 201210, China; wangxinyao@microsate.ac.cn (X.W.); zhengwen@microsate.ac.cn (W.Z.)

**Keywords:** spacecraft detection, lightweight object detection, YOLOv8 enhancement, BiFormer attention, Iterative Attention Feature Fusion (iAFF), information entropy

## Abstract

Non-cooperative spatial target detection plays a vital role in enabling autonomous on-orbit servicing and maintaining space situational awareness (SSA). However, due to the limited computational resources of onboard embedded systems and the complexity of spaceborne imaging environments, where spacecraft images often contain small targets that are easily obscured by background noise and characterized by low local information entropy, many existing object detection frameworks struggle to achieve high accuracy with low computational cost. To address this challenge, we propose YOLO-GRBI, an enhanced detection network designed to balance accuracy and efficiency. A reparameterized ELAN backbone is adopted to improve feature reuse and facilitate gradient propagation. The BiFormer and C2f-iAFF modules are introduced to enhance attention to salient targets, reducing false positives and false negatives. GSConv and VoV-GSCSP modules are integrated into the neck to reduce convolution operations and computational redundancy while preserving information entropy. YOLO-GRBI employs the focal loss for classification and confidence prediction to address class imbalance. Experiments on a self-constructed spacecraft dataset show that YOLO-GRBI outperforms the baseline YOLOv8n, achieving a 4.9% increase in mAP@0.5 and a 6.0% boost in mAP@0.5:0.95, while further reducing model complexity and inference latency.

## 1. Introduction

With the continuous advancement of space technology, human demands for deep space exploration, space resource utilization, and space defense have grown significantly. In response, a growing number of artificial objects—such as satellites, rocket debris, and fragments generated by space operations—have been deployed in Earth orbit [1]. The increasing density of in-orbit objects has substantially elevated the risk of collisions, posing a serious threat to the safety of operational spacecraft [2]. This situation highlights the urgent need to equip in-orbit spacecraft with autonomous environmental sensing capabilities to enable real-time detection and avoidance of potential collision threats, thereby ensuring the safe execution of space missions [3]. Deploying optical sensors on space-based platforms enables the automatic detection and identification of non-cooperative targets, thus providing critical parameters and decision-making support for subsequent On-Orbit Service (OOS) [4,5], Space Situational Awareness (SSA), and Active Debris Removal (ADR) [6] missions.

The core functions of On-Orbit Services (OOS) include in-orbit spacecraft maintenance—such as component replacement, fuel replenishment, and deorbiting—and resource recovery, such as extracting rare materials from defunct spacecraft or acquiring critical operational data [7]. Successful execution of these tasks requires the accurate localization and identification of non-cooperative space targets. “Non-cooperative targets” generally refers to objects such as failed satellites, discarded components, or space debris that lack active communication links and positioning capabilities. Therefore, accurate localization and identification of non-cooperative targets are essential prerequisites for implementing in-orbit servicing operations. For instance, in non-cooperative target capture missions, space robots must first employ visual sensing technologies to detect and localize the target component, followed by identifying a suitable capture region and planning a collision-free trajectory [8].

Space Situational Awareness (SSA) encompasses space object detection, tracking, identification, characterization, event assessment and verification, environmental monitoring, and risk prediction, and serves as the cornerstone of space security [9]. Among these components, space object identification plays a particularly critical role.

In recent years, various sensors have been employed by researchers, including LiDAR [10], time-of-flight (ToF) cameras [11], and monocular or stereoscopic vision systems [12]. Monocular vision systems are particularly favored due to their significant advantages in size, weight, power consumption, and cost [13], and have been implemented in advanced missions such as RemoveDEBRIS [14] and Restore-L [15]. However, variations in scale, viewpoint, and illumination can severely affect the robustness of image feature extraction. Moreover, the onboard computing resources of spacecraft are severely constrained.

The performance of deep neural networks (DNNs) is critically influenced by the quality and scale of training data. Achieving high-precision detection requires large-scale and diverse training datasets. However, acquiring real-world imagery poses considerable challenges, including limited orbital availability, high mission deployment costs, and restricted telemetry bandwidth. To mitigate these limitations, researchers have developed simulation platforms that replicate complex space environments and generate high-quality synthetic imagery as a partial substitute for real data. A representative example is the Stanford University SPEED dataset [16], which contains both real and synthetic images of the Tango satellite under diverse scenarios. Its extended version, SPEED+ [17], further promotes research on cross-domain generalization. In addition, specialized datasets have been constructed for spacecraft component detection and instance segmentation [18]. SPARK, one of the largest datasets for spatial target detection to date, spans multiple spacecraft models and environmental conditions. Its image volume exceeds that of datasets such as SPEED and URSO [19] by over an order of magnitude, offering a robust foundation for training deep learning models. Nevertheless, accurately simulating the complexities of space environments—such as extreme lighting variations, low signal-to-noise ratios, and complex Earth backgrounds—remains a significant challenge. In particular, image artifacts resulting from Earth backgrounds—such as albedo effects, atmospheric scattering, and shadowing—are difficult to model realistically, resulting in substantial domain gaps between synthetic and real images. Consequently, network architectures that perform well in terrestrial applications often fail to generalize to spaceborne scenarios, highlighting the need for algorithmic adaptations tailored to the unique characteristics of space environments.

Compared to traditional methods [20,21], deep neural network-based object detection exhibits superior capabilities in feature extraction, environmental adaptability, transferability, and scalability, making it a widely researched and applied technique in the aerospace domain. For instance, Chen et al. [22] designed a spacecraft component detector based on the Region Proposal Network (RPN), which outperforms traditional sliding window methods, although its real-time performance remains suboptimal. Li et al. [23] proposed SCNN-lite, a single-stage model for the multi-object recognition and localization of non-cooperative spacecraft. However, its ground-based simulation dataset did not sufficiently account for the imaging differences between terrestrial and orbital environments. AlDahoul et al. [24] developed a localization and classification model for non-cooperative space targets based on EfficientDet, achieving promising results in multi-category SSA tasks; however, it lacked optimization for small target detection under complex lighting conditions and failed to incorporate lightweight design constraints aligned with satellite platform limitations. To enhance small target detection, Tao et al. [25] introduced an enhanced spatial feature module and proposed the Space Debris Saliency Detection Network (SDebrisNet). Yuan et al. [26] developed CS-YOLOv5, a context-aware variant of YOLOv5, which detects weak and small space targets using multi-information enhancement modules and data augmentation strategies. Furthermore, Liu et al. [27] introduced the Factor-Ignored Module (FIM) to address issues related to small-sample learning and domain adaptation in the presence of complex Earth backgrounds. When integrated with the YOLO architecture, the FIM substantially improved detection accuracy on simulated datasets. However, the method did not fully address the computational resource constraints inherent to satellite platforms.

Although the above methods perform well in simulated environments, they still face challenges in practical applications:

1. **Illumination variability and extreme lighting**: Spaceborne imagery is frequently acquired under harsh illumination conditions, including strong solar reflections, deep shadows, and high-contrast transitions, which significantly impair target visibility and hinder precise boundary localization.
2. **Limited dataset realism and diversity**: Existing spacecraft detection datasets lack both scale and diversity, particularly in terms of real satellite imagery with varied configurations, orbital conditions, and background complexity, thereby limiting the generalization capabilities of deep learning models.
3. **Small-object detection under constrained resources**: Traditional detection frameworks often fail to balance detection accuracy and inference efficiency, particularly when detecting small-scale spacecraft targets in cluttered or low-SNR environments. Moreover, most existing models are not optimized for deployment on spaceborne edge computing platforms.

To address these challenges, this study proposes **YOLO-GRBI**, a lightweight and robust non-cooperative object detection framework built upon an enhanced YOLOv8 architecture. The proposed design targets three key objectives: robustness to illumination variations, enhanced small-object detection, and real-time performance on resource-constrained platforms. The main contributions of this work are summarized as follows:

1. A non-cooperative spatial target detection dataset containing 9780 real satellite images has been constructed, encompassing a wide range of target scales, viewpoints, and lighting conditions to support robust model training and generalization.
2. To improve detection performance in complex backgrounds and under varying lighting conditions—particularly for small objects—a dynamic sparse attention module (**BiFormer**) is introduced to selectively focus on critical targets while mitigating background interference. Additionally, the C2f module is redesigned by incorporating an Iterative Attention-based Feature Fusion (**iAFF**) mechanism, forming the **C2f-iAFF** module, which enhances cross-layer interactions between deep and shallow features, thereby improving discriminability and robustness in visually complex scenes.
3. To accelerate feature extraction, minimize inference latency, and improve detection efficiency while enabling efficient utilization of computational resources, a **reparameterized ELAN backbone (REB)** has been developed. This backbone improves feature reuse and gradient propagation during training while maintaining low inference latency through branch fusion.
4. To accommodate the limitations of onboard edge computing platforms, a lightweight neck structure has been designed by integrating **GSConv** and **VoV-GSCSP** modules, reducing computational redundancy and enhancing the efficiency of multi-scale feature fusion, without reducing the information entropy generated by convolution.
5. Compared to mainstream YOLO variants and conventional object detection methods, the proposed **YOLO-GRBI** demonstrates notable advantages. By incorporating multiple optimization strategies, the model achieves a balanced trade-off among parameter size, computational complexity, inference speed, and detection accuracy.

The remainder of this paper is organized as follows: Section 2 introduces related work; Section 3 details the overall architecture and key modules; Section 4 introduces the dataset, experimental setup, and evaluation metrics; Section 5 presents performance evaluation and ablation experiment results; Section 6 summarizes the entire paper and discusses limitations and future research directions.

## 2. Related Work

### 2.1. Object Detection Algorithm

Small object detection remains one of the most persistent challenges in the field of object detection. In high-resolution images, small objects often occupy only a minimal pixel area and are easily overlooked during the downsampling process of backbone networks, resulting in significant localization errors and detection failures [28]. Moreover, real-world imaging conditions, such as strong illumination, low-light environments, and cluttered backgrounds, further compound the challenges of small object detection.

To improve detection performance for small objects, researchers have proposed a variety of multi-scale feature fusion strategies, such as FPN [29], PANet [30], and BiFPN [31], which fuse high-level semantic features with low-level spatial details to mitigate localization inaccuracies. The PVswin-YOLOv8s model proposed by Tahir et al. [32] incorporates Swin Transformer and CBAM to enhance detection accuracy for occluded small objects in complex environments. Li et al. [33] designed a small object detection network using the Bi-PAN-FPN architecture combined with the lightweight GhostLockV2 module, thereby reducing model complexity while preserving accuracy. Yang et al. [34] introduced a cascaded sparse query mechanism that utilizes the positional information of small objects in deep features to guide accurate detections by leveraging shallow feature layers. Gao et al. [35] proposed the LACTA algorithm, which achieves lightweight and high-precision detection of small targets such as tomatoes in unstructured environments through structural optimization, demonstrating its applicability in complex real-world scenarios. Song et al. [36] proposed a multi-scale hybrid attention mechanism for small object detection in unmanned aerial vehicle (UAV) images, which effectively captures fine-grained spatial information across multiple scales and enhances detection robustness under complex backgrounds. Su et al. [37] developed MPE-YOLO, an enhanced framework for UAV-based small object detection that employs multi-path feature enhancement and adaptive fusion strategies to improve detection accuracy in aerial imagery containing dense and small-scale targets. Han et al. [38] proposed YOLO-SG, an improved YOLO-based method for detecting small traffic signs in complex scenes, which integrates scale-aware feature enhancement and global context modeling to increase accuracy without significantly raising computational cost. Yi et al. [39] presented an improved YOLO-S model designed for insulator and defect detection. Shen et al. [40] introduced DS-YOLOv8 for object detection in remote sensing images, which incorporates dual-scale feature enhancement and spatial attention to improve the detection of small and densely distributed targets. A recent comprehensive review by Wei et al. [41] systematically summarizes the challenges, mainstream strategies, and future directions of small object detection, highlighting the importance of balancing accuracy, robustness, and computational efficiency across diverse application scenarios.

Although the above methods have made significant progress in terms of accuracy and robustness, most studies have not yet adequately balanced detection accuracy, inference speed, and resource consumption, especially in the context of satellite-based edge computing platform deployment, where their practical feasibility is limited.

### 2.2. Space Target Recognition

Space object recognition constitutes a core technology for space situational awareness, non-cooperative target servicing, collision warning, and deep space mission planning. Its objectives include category classification and localization of satellites, spacecraft, space stations, and their components (e.g., antennas, solar panels, docking rings). Early space target recognition methods primarily relied on handcrafted features and traditional image processing techniques, including contour extraction, edge detection, compressed sensing, and sparse representation [42,43,44]. Although effective under certain conditions, these methods exhibit instability when confronted with complex factors such as target scale variation, illumination disturbances, occlusions, and blurring in deep space imaging. Their limited robustness and generalization capabilities hinder scalability in real-world in-orbit applications.

With the widespread adoption of deep learning, it has been increasingly applied to space target recognition tasks, significantly improving recognition accuracy and efficiency. Zeng and Xia et al. [45] first developed a space target classification model based on a deep convolutional neural network (DCNN) architecture. They utilized STK rendering to generate a multi-class satellite image dataset and employed data augmentation to address the small sample issue, successfully achieving multi-class satellite recognition and demonstrating the potential of deep learning in space image recognition. However, owing to the small scale of targets in space imagery, sparse semantic information, and complex backgrounds, traditional CNN architectures still face challenges in small target recognition. To tackle this, Yang et al. [46] proposed a recognition network combining local component semantics with global features. By extracting local structural information (e.g., solar panels and antennas) and fusing it with overall contour features, the method improved recognition performance for small targets in deep space. However, it struggles to effectively segment components when targets are blurred or severely occluded, resulting in decreased recognition performance.

To address scarce training samples, Yang et al. [47] proposed the D2N4 network tailored for fine-grained few-shot learning. This network mitigates overfitting issues faced by traditional CNNs in few-shot recognition and substantially enhances model generalization capabilities. Additionally, Li et al. [48] constructed satellite 3D models using 3ds Max and applied Cycle-GAN for image style transfer, generating images that more closely resemble real deep-space environments, thereby enhancing model adaptability to complex illumination conditions. They also developed the YSCRM model based on YOLOv5, which improved detection accuracy for components such as solar panels and docking rings. However, this approach primarily targets large, close-range objects and has yet to thoroughly investigate the identification of small, distant targets.

Beyond whole-satellite identification, some studies focus on detecting and segmenting space target components. Chen et al. [22] developed a satellite component detection model based on ResNet-FPN, incorporating dense connections and instance segmentation mechanisms to enhance semantic representation in space object recognition. Zhao et al. [49] combined edge detection with weak semantic supervision to construct a robust edge detection network for non-cooperative satellites. Guo et al. [50] proposed CSAU-Net, a U-Net-based model integrating channel and spatial attention mechanisms, enabling more effective extraction of satellite component boundaries and improving focus on small targets.

Recently, deep neural network-based object detection algorithms have advanced rapidly, particularly the YOLO series, which has garnered widespread attention for its one-stage design, high inference speed, and balanced accuracy. Li et al. [23] developed a lightweight object detection network employing depthwise separable convolutions and residual structures, suitable for resource-constrained in-orbit platforms. Liu et al. [51] introduced the Ghost module and channel compression strategies based on YOLOv5 to design a lightweight satellite component detection algorithm suitable for low-power embedded deployment. Yang et al. [52] proposed a two-stage recognition framework based on MBRT foreground extraction and T-SCNN, separating coarse extraction from fine recognition of target regions, thereby enhancing accuracy and efficiency. Lin et al. [53] proposed a convolutional neural network-based approach for small space target detection, in which guidance information was incorporated to enhance the discrimination of weak targets from background noise. However, systematic comparative studies involving mainstream object detection frameworks, such as YOLOv8 and YOLOv11, remain limited.

## 3. Methods

### 3.1. YOLO-GRBI Model Structure

**Figure 1:**
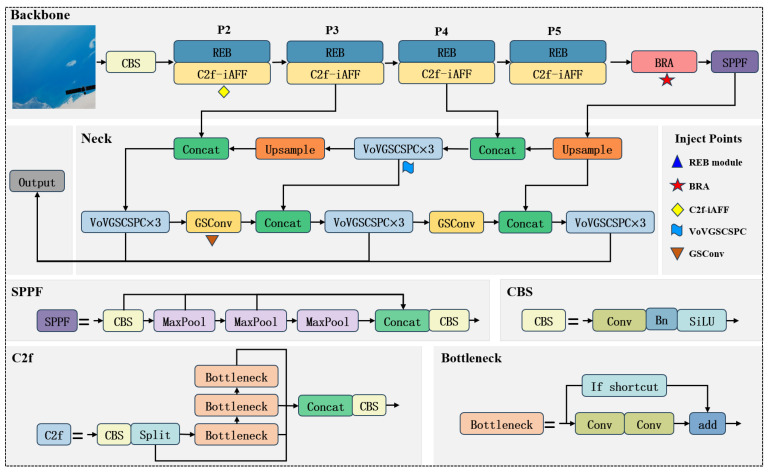
Network Architecture Diagram of YOLO-GRBI.

To improve real-time performance, small-object detection capabilities, and computational efficiency in spacecraft detection tasks, this paper proposes a lightweight enhancement to the YOLOv8 architecture. The proposed improvements emphasize structural reparameterization and attention-guided feature refinement, implemented as illustrated in Figure 1.

First, the Reparameterized ELAN Backbone (REB) is incorporated into the backbone network. Inspired by the ELAN design, REB adopts a multi-branch structure during training to enrich feature diversity and facilitate gradient propagation. During inference, the branches are reparameterized into a single path, reducing computational redundancy while preserving feature expressiveness.

Second, the neck is redesigned using Ghost Convolution (GSConv) and the VoV-GSCSP structure, resulting in a lightweight yet effective feature aggregation module. This Slimneck configuration streamlines the feature fusion process and minimizes redundant computations, thereby enabling efficient multi-scale representation without compromising detection performance.

Third, a dynamic sparse attention mechanism based on BiFormer is integrated into the backbone to enhance the model’s ability to focus on informative regions. By dynamically attending to salient features and suppressing background noise, BiFormer enhances the model’s robustness in cluttered and low-SNR environments, particularly in detecting small or partially occluded objects.

Fourth, to further enhance cross-scale feature interaction, the Iterative Attention Feature Fusion (iAFF) module is integrated into the C2f structure. This module adopts an iterative attention refinement strategy to facilitate the adaptive integration of shallow and deep semantic features, thereby improving the model’s robustness in handling complex spatial structures and fine-grained object variations.

Collectively, these enhancements constitute a compact and efficient detection framework well suited for real-time spacecraft detection under challenging visual conditions. Each module plays a complementary role in improving inference efficiency, feature expressiveness, and detection robustness.

### 3.2. Loss Function in YOLO-GRBI

In the original YOLOv8 framework, the loss function is composed of three main components: the bounding box regression loss $L_{\mathrm{box}}$, the objectness confidence loss $L_{\mathrm{obj}}$, and the classification loss $L_{\mathrm{cls}}$. The objectness and classification branches originally adopt the binary cross-entropy (BCE) loss, which treats each prediction independently and assigns equal importance to all samples. While effective in balanced datasets, BCE tends to be suboptimal in scenarios with severe foreground–background imbalance and a high proportion of easy negative samples—both of which are common in spacecraft detection tasks, particularly when the targets are small and sparsely distributed.

To address this issue, YOLO-GRBI replaces the BCE loss in the objectness and classification branches with focal loss, which introduces a modulating factor to down-weight well-classified samples and focus the training process on hard, misclassified examples. The focal loss is formulated as:

(1) $$L_{\mathrm{focal}}\left(p_{t}\right)=-\alpha_{t}{\left(1-p_{t}\right)}^{\gamma }\log\left(p_{t}\right)$$

where $p_{t}$ represents the model’s estimated probability for the ground-truth class, $\alpha_{t}$ is a balancing factor between positive and negative samples, and γ is the focusing parameter that adjusts the rate at which easy samples are down-weighted.

The final loss function of YOLO-GRBI can thus be expressed as:

(2) $$L_{\mathrm{total}}=L_{\mathrm{box}}+\lambda_{\mathrm{obj}}L_{\mathrm{focal},\mathrm{obj}}+\lambda_{\mathrm{cls}}L_{\mathrm{focal},\mathrm{cls}}$$

where $L_{\mathrm{box}}$ remains the Complete Intersection-over-Union (CIoU) loss for bounding box regression, and $\lambda_{\mathrm{obj}}$ and $\lambda_{\mathrm{cls}}$ are weighting coefficients for the objectness and classification branches, respectively. This modification improves the model’s robustness to class imbalance and enhances its ability to detect small and hard spacecraft targets under challenging imaging conditions.

### 3.3. Neck Structure Optimization

#### 3.3.1. GSConv

To accelerate prediction and enhance the computational efficiency, convolutional neural networks (CNNs) typically transform spatial information into channel-wise representations during the feature extraction process in the backbone. However, this transformation, characterized by a progressive reduction in spatial resolution (width and height) and an increase in the number of channels, can result in the loss of semantic information.

While dense convolution aims to retain inter-channel dependencies to enhance the expressiveness of feature representations, sparse convolution, although efficient in reducing parameters and computational cost, may disrupt inter-channel connectivity and compromise feature integrity.

**Figure 2:**
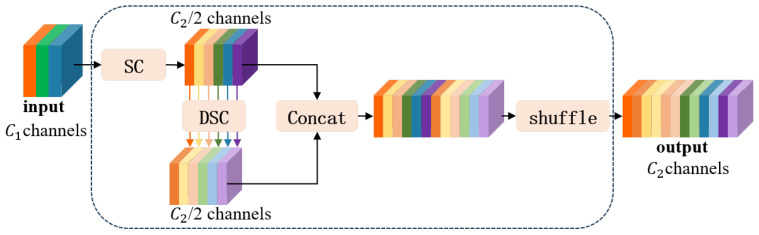
GSConv structure diagram. Different colours indicate distinct convolutional branches and feature fusion paths.

To balance this trade-off between computational efficiency and representational capacity, this study adopts ghost convolution (GSConv), a lightweight operation that combines the advantages of Standard Convolution (SC) and Depthwise Separable Convolution (DSC). GSConv employs a channel shuffle operation to integrate dense information captured by SC into the sparse structure generated by DSC, thereby enhancing channel interactions. The detailed structure is illustrated in Figure 2.

The GSConv operation comprises the following steps: A standard convolution (SC) is first applied for downsampling and initial feature extraction; Depthwise Convolution (DWConv) follows to extract localized spatial features with reduced complexity; the outputs of SC and DWConv are concatenated along the channel dimension; finally, a shuffle operation is performed to mix features across channels, thus promoting inter-channel information exchange.

This design effectively maintains the expressive power of dense convolutions while leveraging the lightweight nature of depthwise operations, resulting in a more efficient and capable convolution mechanism. GSConv significantly enhances both computational performance and the feature extraction capability of the network, making it highly suitable for real-time spacecraft detection tasks.

#### 3.3.2. VoV-GSCSP

**Figure 3:**
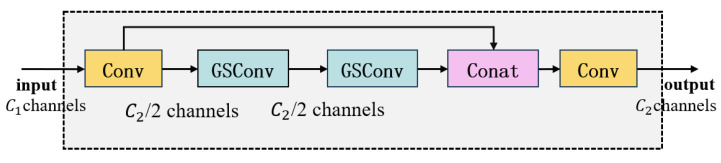
VoV-GSCSP Structure Diagram.

The VoV-GSCSP module extends the capabilities of GSConv by embedding it within an enhanced Cross-Stage Partial Network (CSPNet) framework. Based on the GS Bottleneck structure, VoV-GSCSP adopts a VoVNet-style one-shot aggregation strategy to improve feature reuse and learning efficiency. The detailed structure is illustrated in Figure 3.

It facilitates effective gradient propagation through both main and shortcut branches, which enhances feature diversity and reduces redundancy. By embedding GSConv into the CSPNet structure, VoV-GSCSP achieves substantial reductions in both computational complexity and inference latency while maintaining high detection accuracy.

The incorporation of VoV-GSCSP into the neck network significantly boosts multi-scale feature fusion performance, thereby improving the network’s adaptability to complex spacecraft target scenarios and enhancing small-target detection capability.

### 3.4. Backbone Network Improvements

#### 3.4.1. REB: Reparameterized ELAN Backbone

**Figure 4:**
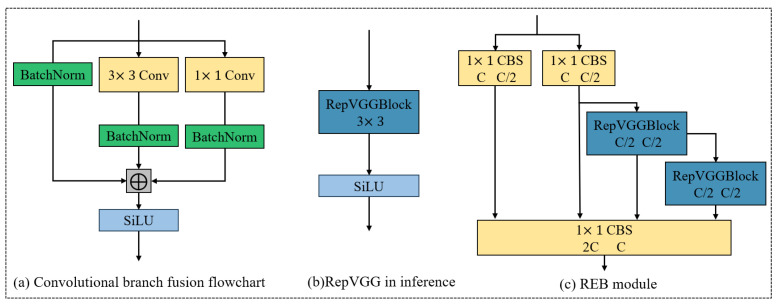
Network structure diagram of REB module.

To enhance both training expressiveness and inference efficiency, the backbone of YOLOv8 is redesigned by integrating the **RepVGG** structure [54] with the **ELAN (Efficient Layer Aggregation Networks)** design philosophy [55], yielding the proposed **REB module**. This architecture combines the multi-branch reparameterization capability of RepVGG with the gradient diversity and feature reuse characteristics of ELAN, as illustrated in Figure 4.

During training, the REB module adopts a multi-branch structure comprising:

- a 3 × 3 convolution,
- a 1 × 1 convolution,
- and an identity mapping path.

These parallel paths enrich feature representations and improve gradient propagation. To support efficient inference, the branches are reparameterized into a single 3 × 3 convolution, thereby significantly reducing model complexity and computational overhead.

Let $W_{3\times 3}$, $W_{1\times 1}$, and I denote the convolutional weights for the 3 × 3, 1 × 1, and identity branches, respectively. Each branch is followed by batch normalization, with parameters $\left(\gamma ,\beta ,\mu ,\sigma^{2}\right)$. The transformation for the 3 × 3 branch is expressed as:

(3) $$\mathrm{BN}\left(x*W_{3\times 3}\right)=\frac{\gamma }{\sqrt{\sigma^{2}+\epsilon }}\left(x*W_{3\times 3}-\mu \right)+\beta$$

This can be folded into an equivalent convolution:

(4) $$\begin{array}{cc} W_{3\times 3}^{\prime } & =\frac{\gamma }{\sqrt{\sigma^{2}+\epsilon }}\cdot W_{3\times 3} \end{array}$$

(5) $$\begin{array}{cc} b_{3\times 3}^{\prime } & =-\frac{\gamma \mu }{\sqrt{\sigma^{2}+\epsilon }}+\beta \end{array}$$

Similar transformations are applied to the 1 × 1 and identity branches, with the 1 × 1 kernels zero-padded to 3 × 3. The final equivalent convolution is obtained via element-wise summation:

(6) $$\begin{array}{cc} W^{\prime } & =W_{3\times 3}^{\prime }+W_{1\times 1}^{\prime }+W_{\mathrm{id}}^{\prime } \end{array}$$

(7) $$\begin{array}{cc} b^{\prime } & =b_{3\times 3}^{\prime }+b_{1\times 1}^{\prime }+b_{\mathrm{id}}^{\prime } \end{array}$$

During inference, the final output is produced through a single convolution followed by a SiLU activation function:

(8) $$y=\mathrm{SiLU}(x*W^{\prime }+b^{\prime })$$

To further improve feature aggregation, the REB module incorporates the ELAN strategy by inserting **two 1 × 1 CBS layers** for feature splitting and aggregation, combined with **cross-stage connections** and **one-shot aggregation modules**. This configuration optimizes both the shortest and longest gradient paths, thereby enhancing convergence speed and model robustness.

Compared with traditional C2f or RepVGG blocks, the REB module achieves:

- enhanced feature reuse through ELAN-style aggregation,
- improved gradient propagation paths,
- stronger representational capacity under constrained depth and parameter budgets,
- and reparameterized inference suitable for lightweight deployment.

This design not only strengthens spatial–spectral coupling but also supports flexible multi-scale fusion, which is essential for accurate detection of small spacecraft in complex orbital environments.

#### 3.4.2. Efficient Attention Mechanism: BRA (Bi-Level Routing Attention)

Spacecraft imagery typically features complex backgrounds and a high density of small-scale targets, posing significant challenges for conventional object detection models. Such models often struggle to effectively suppress background interference, resulting in decreased detection accuracy for small objects. To address this issue, we introduce an efficient dynamic sparse attention mechanism, bi-level routing attention (BRA), into the backbone network to enhance the model’s ability to focus on key regions and suppress irrelevant background information.

The BRA mechanism, implemented through the BiFormer module, employs a query-adaptive routing strategy. Specifically, it filters out key-value pairs that are weakly correlated with the query within coarse-grained regions of the input feature map. This selective filtering allows the model to concentrate computational resources on the most relevant regions, thereby performing attention calculations more efficiently.

From an information-theoretic perspective, the attention weights generated by the BRA module can be interpreted as a probability distribution, where the Shannon entropy reflects the degree of focus in spatial feature selection: A low-entropy distribution indicates that attention is concentrated on a small number of highly discriminative regions, which strengthens local semantic correlation and enhances the feature representation of small targets. In contrast, a high-entropy distribution suggests a more dispersed attention spread, providing richer global context but potentially introducing redundant or noisy information. By regulating the entropy range through its routing mechanism, BRA effectively balances local focus and global perception, enabling the model to suppress background interference while improving feature discrimination, particularly for small or occluded objects.

**Figure 5:**
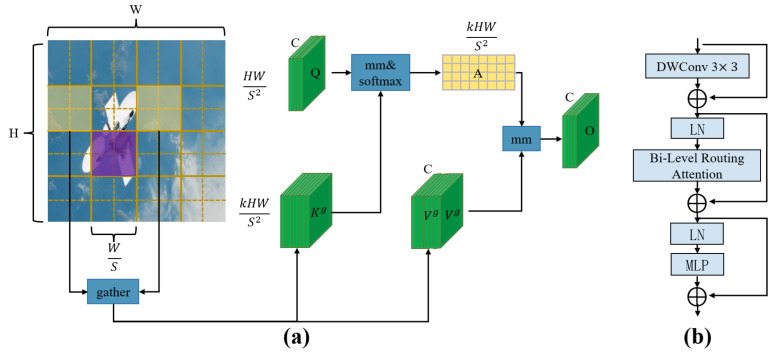
(**a**) Structure of the double layer routing note; (**b**) Structure of the Biformer block.

At the core of BiFormer lies a dynamic sparse attention structure, which enables content-aware and flexible allocation of computational resources. This mechanism dynamically adjusts the attention regions based on the input features, allowing the model to prioritize semantically significant areas. The overall architecture and workflow of the BiFormer module are illustrated in Figure 5a.

As depicted in Figure 5a, the input feature map $X\in R^{H\times W\times C}$ is first partitioned into S×S sub-regions, each containing $\frac{HW}{S^{2}}$ feature vectors, and reshaped into $X_{r}\in R^{S^{2}\times \frac{HW}{S^{2}}\times C}$. Subsequently, linear transformations are applied to obtain the query (Q), key (K), and value (V) matrices as defined in Equations (9)–(11):

(9) $$Q=X_{r}W_{Q}$$

(10) $$K=X_{r}W_{K}$$

(11) $$V=X_{r}W_{V}$$

Next, average pooling is performed within each sub-region to compute the region-level features $Q_{r}$ and $K_{r}\in R^{S^{2}\times C}$. Their dot product is used to construct the adjacency matrix $A_{r}\in R^{S^{2}\times S^{2}}$, which measures inter-region relevance, as shown in Equation (12):

(12) $$A_{r}=Q_{r}K_{r}^{T}$$

The top-*k* most relevant regions for each query region are selected based on $A_{r}$, yielding the routing index matrix $I_{r}\in R^{S^{2}\times k}$, as defined in Equation (13):

(13) $$I_{r}=\mathrm{topkIndex}\left(A_{r}\right)$$

At the fine-grained level, the indexed values $I_{r}\left(i,1\right),I_{r}\left(i,2\right),\ldots ,I_{r}\left(i,k\right)$ are used to gather the corresponding key and value features from *K* and *V*, denoted as $K_{g}$ and $V_{g}$, respectively:

(14) $$K_{g}=\mathrm{gather}\left(K,I_{r}\right)$$

(15) $$V_{g}=\mathrm{gather}\left(V,I_{r}\right)$$

An attention mechanism is then applied to $K_{g}$ and $V_{g}$, and a Local Context Enhancement (LCE) term is added to refine the contextual information. The final output tensor *O* is defined as:

(16) $$O=\mathrm{Attention}\left(Q,K_{g},V_{g}\right)+\mathrm{LCE}\left(V\right)$$

The architecture of the BiFormer module is depicted in Figure 5b. It incorporates depthwise separable convolution (DWConv) to reduce computational cost and parameter count, layer normalization (LN) to accelerate convergence, and a multi-layer perceptron (MLP) to enhance nonlinear feature representation and regulate attention distribution. The addition symbols in the figure represent residual connections, which facilitate information preservation and gradient flow.

To balance detection accuracy with computational efficiency, the BiFormer module is integrated into the backbone of YOLOv8. This design not only satisfies the stringent computational constraints of spacecraft platforms but also significantly enhances feature extraction in complex backgrounds and improves detection performance for small targets.

### 3.5. Feature Fusion Module: C2f-iAFF

**Figure 6:**
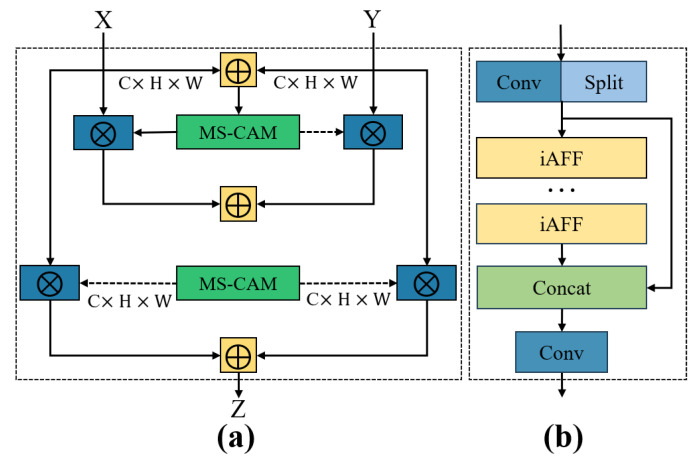
(**a**) Schematic structure of the iAFF model; (**b**) Schematic structure of C2f-iAFF.

The bottleneck structure, initially introduced in ResNet, has been widely adopted in deep neural networks owing to its efficiency in deep feature extraction. In the YOLO series, the C2f module plays a crucial role in enabling lightweight and effective feature representation. To further enhance spacecraft target recognition performance, this study integrates the iAFF (Iterative Attentional Feature Fusion) module into the backbone network, as shown in Figure 6a. The iAFF module introduces an iterative attention-based feature fusion strategy that effectively mitigates information bottlenecks caused by the direct fusion of initial feature maps. Even under constraints of limited model depth and parameter count, iAFF achieves superior feature extraction and enhanced discriminative capabilities.

Based on this refinement, the original C2f module has been architecturally redesigned to form the C2f-iAFF variant, as depicted in Figure 6b. In the revised structure, two convolutional layers—labelled as cv1 and cv2—are initially applied to perform feature extraction and transformation. The resulting feature map is then split into two distinct paths: one is directly forwarded to the output, while the other is passed through a stack of bottleneck layers and subsequently processed by the iAFF module to achieve deeper semantic refinement.

This enhanced module concatenates the outputs of both branches along the channel axis, thereby enriching the diversity of learned features and strengthening representational expressiveness. The integration of the iAFF mechanism further allows the network to dynamically emphasize informative regions while filtering out irrelevant or redundant signals, which in turn boosts the efficiency of multi-scale feature fusion.

To summarize, the proposed C2f-iAFF module significantly elevates the model’s capability in both feature extraction and fusion, ultimately leading to higher detection precision and robustness in spacecraft recognition scenarios. Experimental validation substantiates the module’s effectiveness and its adaptability to complex aerospace environments.

## 4. Experimental Setup

### 4.1. Dataset Construction

The performance of deep learning models heavily depends on the availability of large-scale and high-quality training data. However, publicly available datasets for non-cooperative spatial target detection remain extremely scarce, particularly for small object detection tasks, which pose additional challenges. This scarcity is primarily attributed to the sensitive nature of spaceborne imagery and the strict restrictions placed on its accessibility. Furthermore, the inherent complexity of the space environment exacerbates the difficulty of non-cooperative spatial target detection.

To address these challenges, we constructed the Spacecraft Detection dataset by collecting and annotating 9780 real satellite images from publicly accessible online sources. This dataset is designed to serve as a rich resource for non-cooperative spatial target detection and recognition research.

To better reflect the operational demands of non-cooperative spatial target detection, the constructed dataset incorporates a diverse array of target types representing typical uncooperative space objects. Specifically, it includes decommissioned satellites with surface degradation and structural damage, service modules such as Soyuz and Tianzhou with complex and asymmetric geometries, and spent rocket bodies characterized by irregular shapes and low visual salience. These targets vary significantly in size, shape, texture, and illumination response, presenting high intra-class diversity and visual ambiguity. By integrating such a broad spectrum of non-cooperative spacecraft, the dataset is designed to simulate the diverse and challenging conditions encountered in on-orbit servicing, autonomous rendezvous, and space debris monitoring, thereby providing a more robust benchmark for evaluating detection models under realistic spaceborne scenarios.

The dataset emphasizes the detection and classification of non-cooperative spatial targets, encompassing a wide range of object scales, background complexities, and imaging conditions. The main characteristics are summarized in Table 1. Red frames indicate the target objects that YOLO is expected to detect.

- **Object Scale:** Small objects account for approximately 60% of the dataset, representing the most challenging detection scenario. In addition, medium-sized and large objects each constitute about 20%, ensuring comprehensive coverage across different scales.
- **Background Complexity:** The dataset includes diverse and complex backgrounds, such as deep space, oceans, landmasses, city lights, extreme lighting variations, shadows, and noise interference, significantly increasing the detection difficulty.
- **Target Diversity:** The spacecraft exhibit a wide variety of shapes and structures, including irregular components like solar panels and antennas. Images captured from multiple viewpoints and postures, as well as instances with partial occlusion, are included to enhance task complexity.

To improve the generalization ability of detection models, various data augmentation techniques were employed, including rotation, translation, mirroring, brightness adjustment, and noise injection. These augmentations simulate real-world imaging variations, effectively enriching the diversity of the training data.

For experimental purposes, the dataset was divided into training, validation, and test sets at a ratio of 7:2:1, comprising 6846 training images, 1956 validation images, and 978 test images, respectively. All annotations were performed using the LabelImg tool and saved in COCO format to facilitate model training and evaluation.

### 4.2. Experimental Environment and Parameter Settings

The experiments were conducted on a high-performance computing server, with the detailed hardware and software configurations summarized in Table 2. The server runs a 64-bit Ubuntu 18.04.2 operating system and is equipped with an Intel® Xeon® Gold 6430 CPU and eight NVIDIA GeForce RTX 4090 GPUs. The total system memory is 791 GB, and CUDA 12.4 was utilized for GPU acceleration. All experiments were implemented using Python 3.8 and the PyTorch 2.4.1 deep learning framework.

During training, the input image resolution was set to 640 × 640 pixels, and the batch size was configured to 64. The Stochastic Gradient Descent (SGD) optimizer was used with an initial learning rate of 0.01, a momentum coefficient of 0.937, and a weight decay of 0.0005. The model was trained for 600 epochs. These hyperparameters were determined through extensive experiments to achieve optimal performance. Under this hardware configuration, the training process was efficient and stable, ensuring robust model convergence.

### 4.3. Evaluation Metrics

To thoroughly assess the performance of spacecraft detection, several key metrics were utilized, including precision (P), recall (R), mean average precision (MAP), model size (Params), computational cost (GFLOPs), and processing speed measured in frames per second (FPS). These indicators jointly evaluate both the detection accuracy and operational efficiency of the proposed model.

**Precision (P)** indicates the ratio of correctly predicted positive instances to the total number of predicted positives. It is formally expressed as

(17) $$\mathrm{Precision}=\frac{TP}{TP+FP}$$

**Recall (R)** reflects the model’s ability to correctly identify actual positives, calculated by:

(18) $$\mathrm{Recall}=\frac{TP}{TP+FN}$$

By plotting a Precision-–Recall curve, the **average precision (AP)** for each object category can be determined through the following integral:

(19) $$\mathrm{AP}=\int_{0}^{1}P\left(R\right)\;dR$$

When multiple classes are present in the detection task, the **mean average precision (MAP)** is computed as the average of individual APs across all *N* classes:

(20) $$\mathrm{mAP}=\frac{1}{N}\sum_{n=1}^{N}{\mathrm{AP}}_{n}$$

Unless otherwise specified, all AP scores are reported at an Intersection over Union (IoU) threshold of 0.5, which is a common evaluation criterion in object detection.

In addition to accuracy-related metrics, runtime performance is critical for practical applications. **FPS (Frames per second)** denotes the number of images the model can process per second, offering a direct measure of inference speed.

**Number of parameters (Params)** captures the total count of trainable weights in the network, reflecting its memory footprint and architectural complexity.

Lastly, **computational complexity**, measured in GFLOPs, represents the number of floating-point operations required for a single forward pass, serving as a proxy for computational resource demands during inference.

## 5. Experimental Results

### 5.1. Noise Robustness Evaluation

In real-world spaceborn imaging systems, Gaussian noise commonly arises during image acquisition, encoding, transmission, and compression. To assess the robustness of the proposed detection framework under such conditions, we introduced additive Gaussian noise with zero mean (μ=0) and varying standard deviations (σ=0.05, 0.40, and 0.80) to representative synthetic spacecraft images. The corresponding signal-to-noise ratio (SNR) was computed as

(21) $$\mathrm{SNR}=10\log_{10}\left(\frac{\mu_{\mathrm{signal}}^{2}}{\sigma_{\mathrm{noise}}^{2}}\right)$$

**Figure 7:**
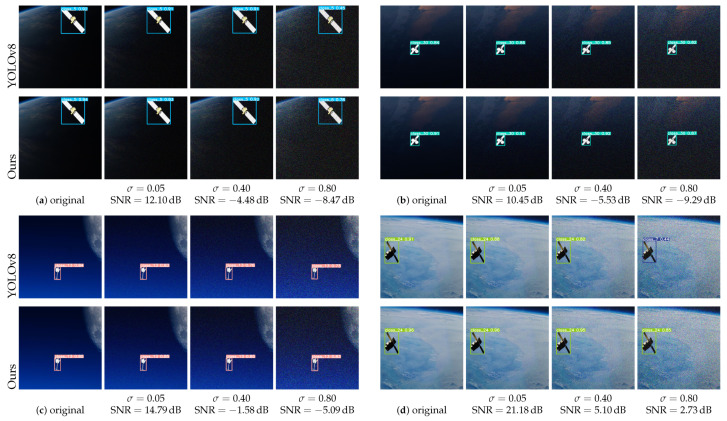
Qualitative comparison of detection results under noisy conditions. Subfigure labels (**a**–**d**) indicate different noise levels and test scenarios. Different colors represent different detected target classes.

Figure 7 shows the qualitative detection results of the baseline YOLOv8 model and the proposed method across four representative image groups, each subjected to progressively increasing noise levels. Within each group, the original and corresponding noisy images are presented side by side for visual comparison.

As illustrated, the baseline model exhibits noticeable performance degradation with increasing noise severity, including missed detections, inaccurate bounding boxes, and false positives. In contrast, the proposed model demonstrates significantly enhanced robustness:

- **Low noise (σ=0.05, SNR ≈ 10–21 dB):** Both models perform reliably; however, the proposed method yields sharper object boundaries and better background suppression.
- **Moderate noise (σ=0.40, SNR ≈−5 to +5 dB):** YOLOv8 predictions become unstable and inconsistent, whereas our method maintains stable, accurate detections while preserving structural and semantic integrity.
- **Severe noise (σ=0.80, SNR ≈−9 to +3 dB):** The proposed approach continues to detect targets with discernible structure, while YOLOv8 often fails to produce meaningful results.

Although such high noise levels are rare in operational space imaging, the results validate the superior noise resilience and generalization capability of our framework.

### 5.2. Ablation Experiments

To comprehensively evaluate the impact of each proposed module on the model’s performance, a series of systematic ablation experiments was conducted. Specifically, the Slimneck module, which integrates GSConv and VoV-GSCSP structures, was first introduced to enhance multi-scale feature fusion efficiency while reducing the overall parameter count. Subsequently, modules including the Reparameterized ELAN Backbone (REB), BiFormer, and c2f-iAFF and an alternative classification loss function (focal loss) were incrementally incorporated to evaluate their individual and combined contributions in terms of detection accuracy, parameter efficiency, computational complexity (GFLOPs), and inference speed. The results are summarized in Table 3, highlighting the performance variations induced by the integration of each module.

The introduction of the Slimneck module improved the precision from 83.4% to 84.1% and the recall from 75.2% to 79.8%, while the mAP (0.5) increased from 84.0% to 85.4%. Additionally, the parameter count decreased from 3.01 M to 2.70 M, and GFLOPs reduced from 8.2 to 7.1, indicating enhanced efficiency with minimal computational overhead.

When incorporating the REB module independently, the precision significantly increased to 84.1%, the recall to 80.5%, and the mAP (0.5) reached 86.1%, with GFLOPs remaining comparable at 8.1. These results suggest that REB’s multi-branch training structure and reparameterized inference architecture enhance feature expressiveness while maintaining computational efficiency.

The combination of Slimneck and REB led to further improvements, achieving a precision of 87.1%, a recall of 81.0%, and a MAP (0.5) of 87.6%, while maintaining a low parameter count (2.75M) and reduced GFLOPs (7.2). This demonstrates an optimal balance between detection accuracy and computational cost.

Integrating the BiFormer module yielded additional gains, with the precision and recall improving to 84.7% and 80.8%, respectively, and the MAP (0.5) increasing to 85.8%. Its dynamic sparse attention mechanism enhances the model’s capability to capture long-range dependencies and suppress irrelevant background information, making it especially effective in cluttered or complex scenes.

The c2f-iAFF module, when added individually, improved the mAP (0.5) to 85.6% while maintaining relatively stable computational overhead. Its iterative attention-based fusion strategy facilitates fine-grained multi-scale feature aggregation, thereby improving feature discriminability and stability.

The combination of BiFormer and c2f-iAFF led to further improvements, achieving a precision of 85.0%, a recall of 80.1%, and a MAP (0.5) of 86.3%. BiFormer and c2f-iAFF provide complementary attention capabilities across global and local scales. While BiFormer is adept at modeling long-range dependencies and capturing global context through sparse attention, c2f-iAFF enhances local consistency and detail preservation via iterative feature fusion and channel–spatial recalibration. This global–local attention cooperation enables the model to achieve both coarse-level contextual understanding and fine-level spatial precision, which is particularly advantageous for detecting small, irregular, or partially occluded spacecraft.

The best overall performance was obtained by integrating all four modules, resulting in a precision of 90.9%, a recall of 81.3%, and a MAP (0.5) of 88.5%, while keeping the parameter count at only 2.90 M and GFLOPs at 7.9. These findings validate the effectiveness of the collaborative module design, in which each component contributes complementary benefits. When integrated together, all four modules establish a functionally diverse yet hierarchically coherent architecture: REB strengthens the semantic depth and feature diversity at the backbone level; Slimneck compresses and refines multi-scale information in the neck; BiFormer ensures global attention and background suppression; and c2f-iAFF fine-tunes local details and spatial alignment at the prediction stage. This hierarchical division of labor results in a structurally efficient and functionally robust detection pipeline. Such a multi-level collaborative design significantly improves the model’s adaptability to non-cooperative or degraded imaging conditions in space missions, including scenarios with strong illumination variation, low signal-to-noise ratio, and densely cluttered backgrounds.

Finally, replacing the conventional binary cross-entropy loss with focal loss on top of the full model further increased the recall from 81.3% to 81.8% and MAP (0.5) from 88.5% to 88.9%, while keeping parameters and GFLOPs unchanged. This ablation study comprehensively demonstrates the contributions and synergies of each module to model lightweighting, accuracy enhancement, and computational efficiency. The proposed multi-module collaborative optimization strategy not only improves the model’s adaptability to complex targets but also strengthens its practical applicability in spacecraft detection tasks, particularly in challenging scenarios involving small targets, cluttered backgrounds, strong light variations, and degraded image conditions. The complementary functions across modules—in backbone expressiveness, neck-level efficiency, global-local attention modeling, and multi-scale feature alignment—collectively enable robust and real-time spacecraft detection in dynamic space environments.

### 5.3. Comparative Experiment of Different Models

To validate the effectiveness and advancements of the proposed model for spacecraft target detection, a comparative study was conducted involving several mainstream object detection models. The compared models include various versions of the YOLO series (YOLOv3-tiny, YOLOv5s, YOLOv6, YOLOv8n, YOLOv11, YOLOv12, CS-YOLO, and LACTA), a representative transformer-based model (RT-DETR), and classic CNN-based architectures (e.g., ResNet50 and ResNet101) [56]. Among them, CS-YOLO and LACTA are YOLO-based detection algorithms specifically enhanced for small object detection. The evaluation metrics included precision, recall, mAP@0.5, mAP@0.5:0.95, parameter count, model size, and inference time. The results are summarized in Table 4.

As shown in Table 4, the proposed model outperforms other competitors across several key metrics, particularly in terms of precision, mAP@0.5, and mAP@0.5:0.95. Compared with the baseline YOLOv8n model, which achieved 90.8% precision and 88.9% mAP@0.5, the proposed model improves precision by 7.4 percentage points and mAP@0.5 by 4.9 percentage points, while maintaining a lightweight architecture and faster inference speed.

Although YOLOv3-tiny has a compact model size, its detection accuracy remains suboptimal, particularly for small object detection, achieving only 80.4% precision and 68.3% recall. YOLOv5s demonstrates good precision but exhibits a high parameter count of 7.05 M and a longer inference time of 11.8 ms, rendering it less suitable for resource-constrained aerospace applications. YOLOv6 exhibits relatively low parameter complexity (4.23 M) but does not outperform the other models in terms of accuracy. Although YOLOv11 and YOLOv12 achieve improvements in model size and inference speed, they still fall short in overall accuracy compared with the proposed model. Compared with CS-YOLO, which is optimized for weak and small space object detection, the proposed YOLO-GRBI achieves improvements of 1.4% in mAP50, 1.7% in mAP50-95, and 1.1% in Recall, while reducing the number of parameters by 59.0%, the model size by 59.3%, and the inference time by 1.1 ms, demonstrating a better trade-off between accuracy and efficiency. Compared to LACTA, a lightweight detector tailored for small object detection, YOLO-GRBI improves mAP50, mAP50-95, and recall by 2.0%, 2.6%, and 1.6%, respectively. Although the parameter count is slightly higher than that of LACTA (increased by 1.7 M), YOLO-GRBI delivers superior detection accuracy and faster inference while maintaining a compact model size (only 1.7 MB larger), making it more suitable for resource-constrained deployment scenarios.

Transformer-based RT-DETR and CNN-based ResNet models exhibit competitive accuracy in certain metrics; however, their high parameter counts, large model sizes, and extended inference times render them unsuitable for deployment on spaceborne platforms with limited computational resources.

In summary, the proposed model achieves 90.8% precision, 88.9% mAP@0.5, and 76.2% mAP@0.5:0.95, with only 2.90 M parameters and an inference time of 2.4 ms, effectively balancing detection performance, computational efficiency, and lightweight design. These results demonstrate the strong potential of the proposed model for spacecraft detection tasks.

### 5.4. Visualization Experiments

To further assess the practical detection performance of the proposed model on real spacecraft imagery, we conducted a series of visualization experiments addressing typical challenges such as small object detection, complex background adaptation, illumination robustness, and multi-scale and multi-pose target recognition, with comparisons made against the baseline YOLOv8 model.

**Figure 8:**
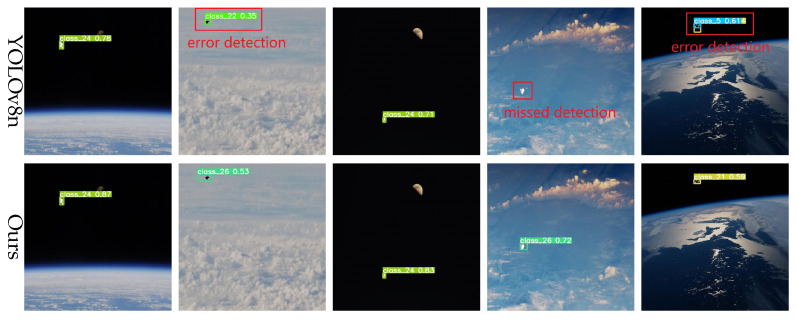
Small target detection results.

In spacecraft detection tasks, particularly in space environments, many targets are relatively small, presenting significant challenges to detection models. To evaluate our model’s performance in small object detection, representative scenarios were selected for visual comparison. As illustrated in Figure 8, the YOLOv8 baseline model exhibits noticeable localization errors for small objects, often missing or falsely detecting targets. In contrast, our improved model accurately localizes these targets, significantly reducing false detections. Furthermore, it effectively suppresses background noise and demonstrates significant advantages in small object detection.

**Figure 9:**
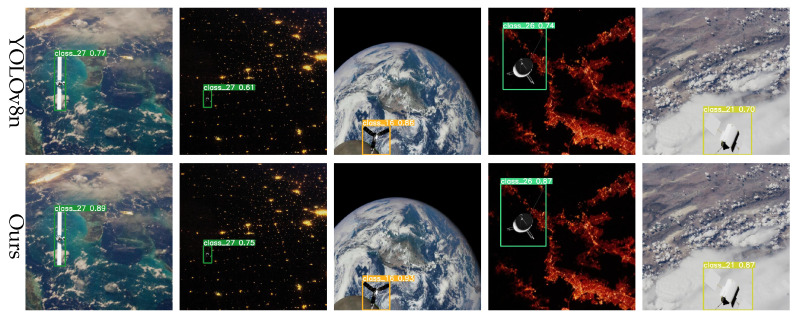
Detection results in complex backgrounds.

Background complexity is another critical factor affecting detection accuracy, particularly when targets are presented under conditions such as illumination variation, occlusion, or other challenging backgrounds. To evaluate robustness under these conditions, we tested on images characterized by high noise levels and complex scenes. The results, illustrated in Figure 9, show that the YOLOv8 baseline model struggles under complex backgrounds. By contrast, our improved model, enhanced with the BiFormer module’s dynamic sparse attention mechanism, adaptively focuses on salient regions, thereby reducing background interference and significantly enhancing detection performance in complex scenarios.

**Figure 10:**
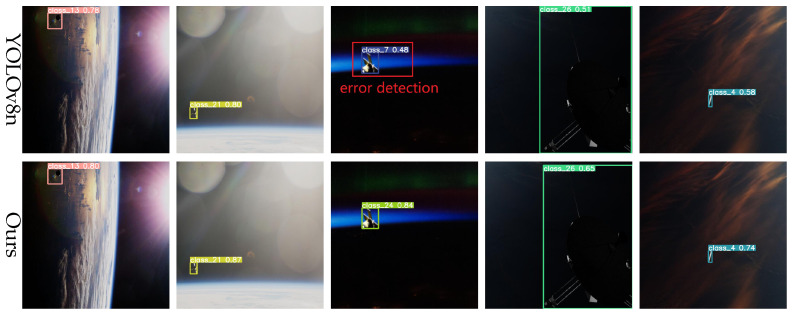
Detection results under light change.

In space, lighting conditions can change dramatically, particularly during transitions into or out of the Earth’s shadow. To evaluate robustness under such conditions, a test set simulating strong illumination variations was designed. As shown in Figure 10, the YOLOv8 baseline model exhibits bounding box inaccuracies and even misses detections under extreme illumination changes. In contrast, the proposed model maintains accurate detections and robust boundary fitting under the same conditions, demonstrating superior adaptability to illumination variations.

**Figure 11:**
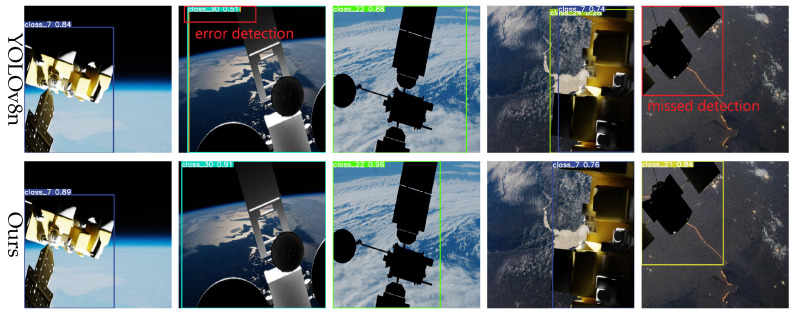
Multi-scale and multi-attitude target detection results.

The pose and scale of spacecraft vary substantially in space, often involving significant rotations. To evaluate the model’s capability for multi-scale and multi-pose detection, we conducted tests on images featuring spacecraft with diverse poses and scales. As shown in Figure 11, the YOLOv8 baseline exhibits inaccurate localization and substantial errors under certain viewpoints. Conversely, the proposed model accurately detects targets across varying poses and scales, maintaining high precision and robustness even under large rotations and scale variations.

### 5.5. Generalization Experiments

To further evaluate the generalization capability of the proposed model, we performed experiments using several widely adopted public spacecraft datasets. A brief overview of each dataset is provided below:

- **Spacecraft Dataset** [18]: This dataset, introduced by Dung et al., is designed to support spacecraft detection, part recognition, and instance segmentation. The dataset integrates both real and synthetic images. It contains 3117 high-resolution images (1280 × 720) with annotations including bounding boxes, instance masks, and part masks. Preprocessing steps were applied to eliminate redundancy, and segmentation masks were refined using Polygon-RNN++.
- **SPEED Dataset** [16]: The SPEED dataset was created for the Satellite Pose Estimation Challenge (SPEC) organized by Stanford University and the European Space Agency (ESA). It includes 14,998 synthetic and 300 real grayscale images at high resolution, facilitating the development and evaluation of deep learning models for spaceborne vision tasks.
- **SPEED+ Dataset** [17]: As an extension of the original SPEED dataset, SPEED+ provides 60,000 synthetic training images and 9531 simulated spacecraft images. This augmentation offers a wider variety of poses and environmental conditions, making it suitable for evaluating the robustness of on-board AI algorithms.
- **URSO Dataset** [19]: The Unreal Rendered Spacecraft On-Orbit (URSO) dataset provides realistic RGB images and depth maps rendered using Unreal Engine 4. It focuses on scenarios such as space rendezvous, docking, and debris removal. The dataset includes 5000 images at 1080 × 960 resolution, covering randomly sampled viewpoints in Low Earth Orbit. Included spacecraft models feature Soyuz and Dragon.

These datasets encompass diverse spacecraft shapes, poses, illumination conditions, and sensor characteristics, offering a comprehensive benchmark for evaluating model generalization in realistic space scenarios.

To validate the generalization performance of the proposed model, we conducted experiments on the Spacecraft Dataset and compared the results with several mainstream object detection models. To ensure a fair comparison, all models were trained using identical strategies and hyperparameter settings as those applied in the original experiments. The performance comparison results are summarized in Table 5.

The results demonstrate that our improved YOLOv8-based model achieves superior performance on the generalization dataset, attaining a mAP@0.5 of 96.6% and a mAP@0.5:0.95 of 89.5%, thereby outperforming all other competing detection models. It remains lightweight, with only 2.90 million parameters—fewer than other high-performing models—highlighting its efficiency in balancing accuracy and computational cost.

**Figure 12:**
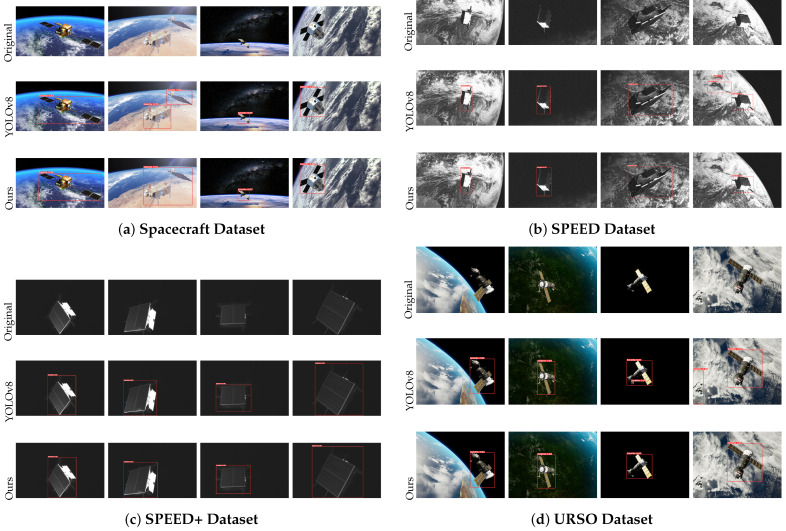
Detection performance comparison across four public spacecraft datasets: (**a**) Spacecraft Dataset, (**b**) SPEED, (**c**) SPEED+, and (**d**) URSO. Each group shows visual results from original images, YOLOv8, and our model.

To provide a clearer and more intuitive comparison of model performance in generalization scenarios, we visualized and compared the detection results of YOLOv8n and our proposed model across multiple spacecraft datasets. Figure 12 illustrates the detection outputs on the Spacecraft Dataset, SPEED, SPEED+, and URSO datasets.

Notably, the SPEED and SPEED+ datasets comprise real satellite detection data, representing complex and diverse space environments. Experimental results demonstrate that our proposed model generates more accurate and precisely aligned bounding boxes under varying viewpoints, scales, and complex backgrounds, while effectively suppressing background noise. Compared to YOLOv8n, our model exhibits superior robustness and generalization capability.

In summary, our proposed model not only achieves excellent performance on proprietary datasets but also maintains stable adaptability across various complex space scenarios, significantly enhancing the practical value and reliability of spacecraft detection tasks.

## 6. Discussion

In real-world space payload deployments, deep learning models must accommodate a range of hardware and environmental constraints. Radiation effects in space, such as single-event upsets (SEUs), can compromise the stability of memory and computational units, while thermal cycling and temperature gradient fluctuations can alter hardware operating conditions, thereby increasing inference uncertainty. Consequently, selecting an appropriate embedded computing platform is critical. The NVIDIA Jetson platform series is specifically designed to accelerate machine learning applications by leveraging GPU architectures, enabling complex deep learning models to run directly on-device. Their low-latency, high-efficiency inference capabilities have led to widespread adoption in robotics, autonomous vehicles, and industrial automation.

Specifically, the Jetson Orin Nano integrates a multi-core ARM A78AE CPU (up to six cores, clocked at 1.5 GHz) with an NVIDIA GPU (8 SMs, clocked at up to 0.625 GHz), providing sufficient computational power for processing complex sensor data while maintaining a maximum power consumption of just 15 W, making it highly suitable for space applications with strict power constraints. Modern space missions increasingly rely on in-situ data extraction, imposing stringent requirements on processing platforms for low latency and high reliability. Research [57] indicates that the Orin Nano and its series products (such as the Orin NX and Xavier NX) can withstand a total ionizing dose (TID) of at least 20 krad, demonstrating excellent radiation resistance in low Earth orbit (LEO) missions; although CPU clock frequency may decrease at higher doses (50 krad), their overall radiation tolerance renders them an ideal choice for space applications.

In extreme space-based deployment scenarios, such as CubeSats or microsatellites, limited computational power and storage capacity necessitate highly lightweight deep learning models. Model compression techniques, including quantization and pruning, effectively reduce the number of parameters and computational complexity, thereby lowering storage requirements and power consumption.

Pruning enhances computational efficiency by eliminating redundant or insignificant neural network parameters, thereby substantially reducing model size without markedly compromising accuracy. It is well-suited for deployment environments with limited computational resources or requiring real-time responses. This technique has been shown to significantly reduce memory usage and inference time and, in some cases, maintain or even surpass the performance of the original network. Depending on available computational resources and operational complexity, pruning can be applied during or after model training [58]. Pruning is primarily categorized into unstructured and structured approaches. Unstructured pruning removes sparse connections within the network by setting individual weights below a predetermined threshold (based on weight magnitude or gradient) to zero, though its effect on inference latency is limited. In contrast, structured pruning eliminates entire weight groups (such as convolutional filters, kernels, or channels), effectively reducing forward propagation computational demands and substantially improving inference speed.

Quantization is the process of reducing the precision of neural network parameters from a higher bit width (n bits) to a lower bit width (m bits) (n > m) [59]. CNN training typically employs 32-bit floating-point (fp32) precision, with common quantization targets including 16-bit floating-point (fp16) or 8-bit integer (int8). The primary advantage of quantization is improved inference speed, as integer operations generally exhibit lower computational complexity than floating-point operations, and low-precision numerical representations are more efficient, significantly reducing latency, energy consumption, and resource usage, albeit with minor accuracy loss. Quantization strategies are primarily divided into post-training quantization (PTQ) and quantization-aware training (QAT). PTQ directly quantizes pre-trained floating-point models, often incorporating layer fusion (e.g., convolution, batch normalization, and activation layers) to minimize potential quantization errors and employing calibration datasets to estimate the dynamic range of weights and activations, thereby enhancing quantized model accuracy [60]. QAT simulates quantization during model training by inserting “fake quantization” nodes into weights and activations while maintaining backpropagation in floating-point precision, enabling the model to adapt to precision loss introduced by quantization. Nonetheless, QAT generally necessitates additional training epochs and exhibits sensitivity to training hyperparameters.

## 7. Conclusions

This study proposes an enhanced object detection network, YOLO-GRBI, which features a reparameterized ELAN backbone (REB), incorporates a dynamic sparse attention module (BiFormer) and a C2f_iAFF module, and refines the neck structure by integrating GSConv and VoV-GSCSP modules. The proposed model demonstrates enhanced stability and stronger attention to critical object features, while simultaneously reducing model complexity and improving inference speed.

Extensive experiments on a custom spacecraft dataset demonstrate that the model achieves outstanding recognition performance in complex spatial environments, particularly for multi-scale and small objects. Even under Gaussian noise perturbation, YOLO-GRBI maintains strong detection performance. The enhanced model effectively detects targets of various sizes and categories, significantly improving recognition accuracy across diverse object types.

Despite these promising results, two limitations remain. First, the current study evaluates the proposed model solely in the context of non-cooperative spacecraft detection. Although the designed architecture addresses common challenges such as small object visibility, low information entropy, and resource constraints, its generalizability to other vision tasks—such as ecological monitoring, industrial defect detection, or UAV-based object tracking—has yet to be systematically investigated. Second, while YOLO-GRBI is optimized for deployment on resource-limited platforms, this work lacks practical validation on real embedded hardware systems (e.g., Jetson Nano, FPGA), which is essential for assessing actual runtime performance and energy efficiency in operational settings.

In future work, we aim to address these limitations by extending the evaluation of YOLO-GRBI to broader application domains and conducting real-world deployment experiments on embedded systems. We also plan to further enhance the model’s lightweight characteristics using advanced compression techniques such as pruning and quantization and to incorporate fine-grained component-level reasoning to improve recognition accuracy under extreme orbital imaging conditions.

## Figures and Tables

**Table 1:** Characteristics and Descriptions of the Dataset.

Specificities	Description	Images			
Multiscale	Extreme target scale variations with a high ratio of small targets	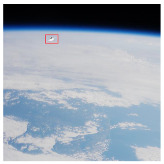	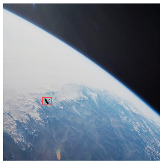	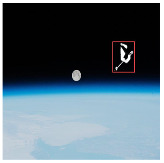	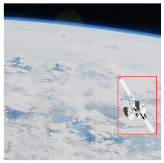
Complex context	Deep space and Earth surface variations	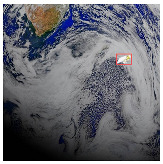	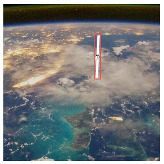	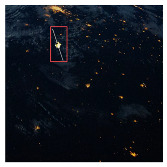	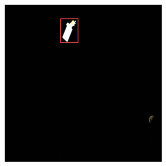
Sunlight	Drastic lighting variations (glare, shadows)	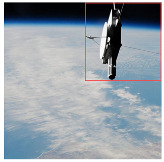	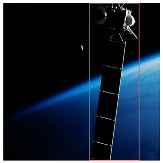	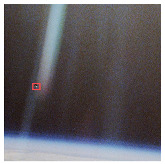	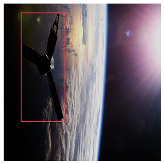
Multi-perspective	Multi-attitude spacecraft (rotation, occlusion)	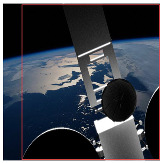	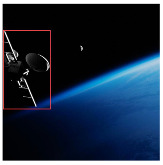	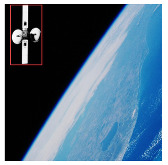	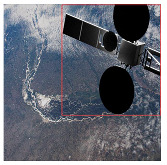
Variety of target patterns	Components of diverse configurations	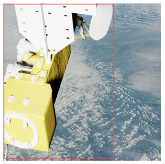	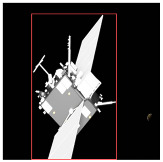	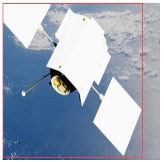	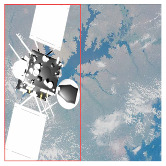

**Table 2:** Detailed configuration information of the experimental environment.

System Environment	Ubuntu 18.04.2 64Bit
CPU	Intel(R) Xeon(R) Gold 6430
GPU	NVIDIA GeForce RTX 4090 x8
Memory	791 GB
CUDA	12.4
Python	3.8
Pytorch	2.4.1

**Table 3:** Results of ablation experiments, where A1: Slimneck, A2: REB, A3: BiFormer, A4: c2f-iAFF, A5: Focal Loss.

Number	A1	A2	A3	A4	A5	Precision	Recall	mAP (0.5)	Params (M)	GFLOPs
1						83.4%	75.2%	84.0%	3.01	8.2
2	✓					84.1%	79.8%	85.4%	2.70	7.1
3		✓				84.1%	80.5%	86.1%	3.05	8.1
4			✓			84.7%	80.8%	85.8%	3.09	8.5
5				✓		84.9%	78.9%	85.6%	3.12	8.4
6	✓	✓				87.1%	81.0%	87.6%	2.75	7.2
7	✓		✓			85.4%	81.5%	86.9%	2.78	7.6
8	✓			✓		85.2%	80.6%	86.5%	2.80	7.4
9			✓	✓		85.0%	80.1%	86.3%	3.19	8.7
10	✓	✓	✓			88.2%	82.1%	87.8%	2.85	7.6
11	✓		✓	✓		87.6%	81.0%	87.9%	2.88	7.8
12	✓	✓	✓	✓		90.9%	81.3%	88.5%	2.90	7.9
13	✓	✓	✓	✓	✓	90.8%	81.8%	88.9%	2.90	7.9

**Table 4:** Comparative experimental results of different models.

Model	Precision	Recall	mAP (0.5)	mAP(0.5:0.95)	Params (M)	Model (MB)	Detection Time/ms
YOLOv8n	83.4%	75.2%	84%	70.2%	3.01	6.03	2.7
YOLOv3-tiny	80.4%	68.3%	77.6%	62%	12.13	17.3	2.2
YOLOv5s	86%	75.1%	84.4%	70.5%	7.05	14.5	11.8
YOLOv6	81.9%	76%	82.1%	68.4%	4.23	8.41	3.0
YOLOv11	88.1%	75.2%	84.7%	71.3%	2.58	5.31	1.6
YOLOv12	87.4%	80.1%	87.3%	74.2%	2.55	5.38	1.5
CS-YOLO	89.6%	80.7%	87.5%	74.5%	7.08	14.7	3.5
LACTA	89.1%	80.2%	86.9%	73.6%	1.20	2.60	2.7
RT-DETR-l	80.7%	72%	76.6%	62.5%	32.01	64.0	3.1
RT-DETR-x	82%	70.5%	76.4%	62.7%	65.49	131.0	5.0
ResNet50	87.9%	71.1%	79.1%	64.4%	41.96	83.9	3.5
ResNet101	85.6%	73.8%	79.7%	65.5%	60.92	121.8	4.5
Ours	90.8%	81.8%	88.9%	76.2%	2.90	5.99	2.4

**Table 5:** Results of generalization experiments on the Spacecraft Dataset.

Model	Precision	Recall	mAP@0.5	mAP@0.5:0.95	Params (M)
YOLOv3-tiny	89.0%	86.9%	91.8%	71.9%	12.13
YOLOv5s	95.3%	90.0%	94.9%	86.9%	7.05
YOLOv6	95.8%	89.7%	95.7%	87.8%	4.23
YOLOv8n	94.8%	90.9%	95.7%	88.4%	3.01
CS-YOLO	96.0%	88.5%	95.6%	88.1%	7.08
LACTA	95.5%	88.2%	95.3%	87.5%	1.20
YOLOv11	95.8%	90.1%	95.9%	87.9%	2.58
YOLOv12	95.7%	90.4%	96.2%	87.7%	2.55
Ours	96.8%	88.9%	96.6%	89.5%	2.90

## Data Availability

All experimental data supporting the findings of this study are included in the article. Additional datasets or related information can be obtained by contacting the first or corresponding author upon reasonable request.
